# Diversity profiling of microbiomes associated with selected alpine plants and lichens from Mt. Suisho, Japan

**DOI:** 10.1128/mra.01270-23

**Published:** 2024-02-16

**Authors:** Ryosuke Nakai, Shouno Munekawa, Yoshinori Murai, Yoshihito Ohmura, Kenichiro Tani

**Affiliations:** 1 Bioproduction Research Institute, National Institute of Advanced Industrial Science and Technology (AIST), Sapporo, Hokkaido, Japan; 2 Department of Botany, National Museum of Nature and Science, Tsukuba, Ibaraki, Japan; 3 Department of Geology and Paleontology, National Museum of Nature and Science, Tsukuba, Ibaraki, Japan; Queens College, Queens, New York, USA

**Keywords:** alpine, plant, lichen, microbiome

## Abstract

We report 16S rRNA gene amplicon data for the microbiomes in selected alpine plants (genera *Artemisia*, *Parnassia*, and *Phyllodoce*) and lichens (genera *Cladonia* and a mixture of *Miriquidica* and *Rhizocarpon*) from Mt. Suisho, Japan. Most of these samples were dominated by *Pseudomonadota*, while some contained the rarely cultivated phylum *Vulcanimicrobiota* (*Candidatus* Eremiobacterota/WPS-2).

## ANNOUNCEMENT

Recent culture-independent omics approaches have unraveled the phylogenetic diversity and novelty of microbiomes associated with plants and lichens (1, 2). Here, we provide baseline data on the microbiome in the alpine region of Japan.

Different alpine plants (genera *Artemisia*, *Parnassia*, and *Phyllodoce*) and lichens (genera *Cladonia* and a mixture of *Miriquidica* and *Rhizocarpon*) were collected at an altitude of 2,668 m on Mt. Suisho, Japan (36.424 N 137.598 E). The samples were transferred to the laboratory and stored at –80°C until further analysis; as for the plant samples, only the roots were excised and stored. First, soil surrounding the plant roots and lichens collected from rocks were used as samples for microbiome analysis. The subsequent experiments were conducted in duplicate per sample. Microbial DNAs were extracted using Extrap Soil DNA Kit Plus v.2 (BioDynamics Laboratory Inc.), following the manufacturer’s instructions with no modifications. The first PCRs targeting the 16S rRNA gene V4–V5 regions were amplified using primers 515F-Y (ACACTCTTTCCCTACACGACGCTCTTCCGATCT-GTGYCAGCMGCCGCGGTAA) and 926R (GTGACTGGAGTTCAGACGTGTGCTCTTCCGATCT-CCGYCAATTYMTTTRAGTTT) (3), following the 16S Metagenomic Sequencing Library Preparation protocol (provided by Illumina Inc.). The second PCRs were performed to attach adaptor sequences using the following primers: forward primer (AATGATACGGCGACCACCGAGATCTACAC-index2-ACACTCTTTCCCTACACGACGC) and reverse primer (CAAGCAGAAGACGGCATACGAGAT-index1-GTGACTGGAGTTCAGACGTGTG). The PCR products were purified with AMPure XP (Beckman Coulter). After purification, paired-end sequencing was performed on an Illumina MiSeq platform using a MiSeq Reagent Kit v.3 (Illumina). The reads containing the primer sequences were isolated using the fastx_barcode_splitter of the FASTX-Toolkit v. 0.0.14 (http://hannonlab.cshl.edu/fastx_toolkit/). The primer sequences were then trimmed with fastx_trimer from the FASTX-Toolkit. Low-quality reads (*Q* score of <20) and short reads (<130 bp) and their read pairs were removed with Sickle v.1.33 with the options of -q 20 and -l 130 (https://github.com/najoshi/sickle). The remaining reads were assembled using FLASH v.1.2.11 with the options of -r 280, -f 410, and -m 10 (4). The assembled reads were processed using QIIME 2 v.2023.2 (5) with Greengenes v.13.8 (6), employing denoising and chimera checks with the DADA2 plugin (7).

**Fig 1:**
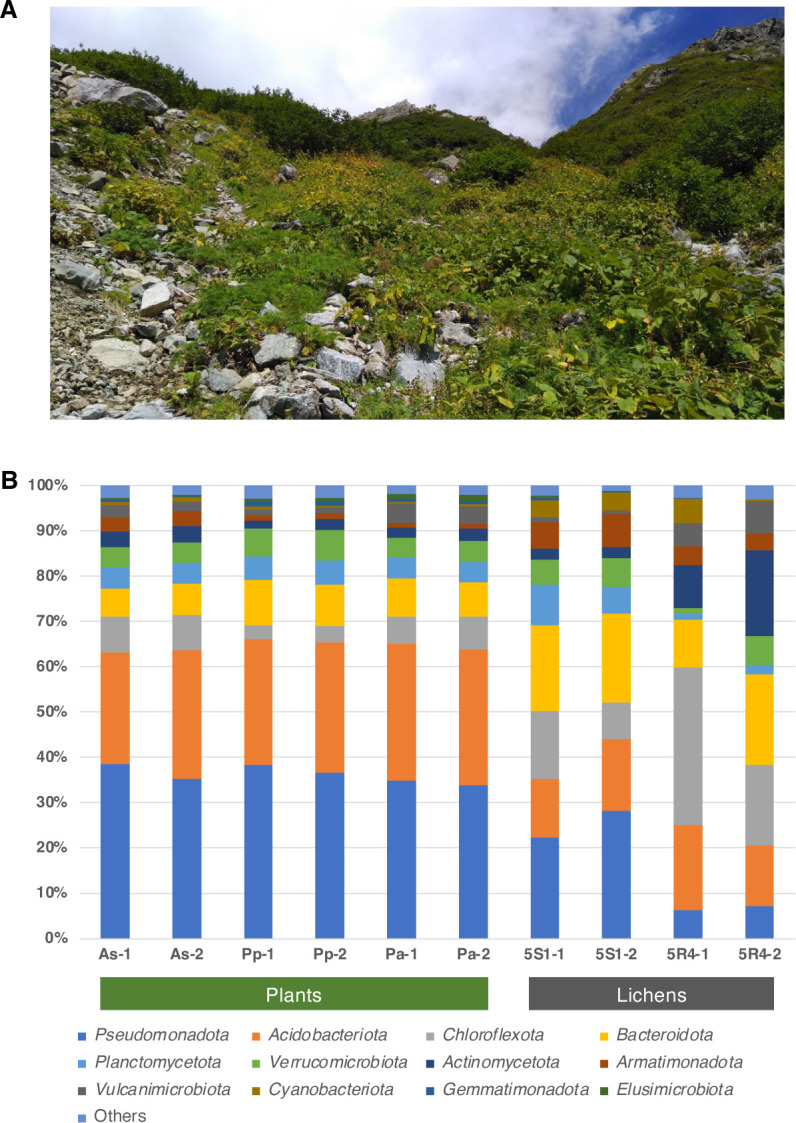
Relative abundance of the taxonomic composition of the microbiomes derived from selected alpine plants and lichens. **(A**) Image of the sampling point at Mt. Suisho. (**B**) Phylogenetic lineages containing more than 1% relative abundance in at least two samples are indicated, and others are grouped together as "others." The description of the phylum names followed the List of Prokaryotic names with Standing in Nomenclature (https://lpsn.dsmz.de/).

Microbiome amplicon data analyzed in this study are shown in Table 1 and Fig. 1. Note that the sample IDs of As, Pp, and Pa in the alpine plant samples correspond to *Artemisia sinanensis*, *Parnassia palustris* var. *tenuis*, and *Phyllodoce aleutica* identified by our co-author Y. Murai, respectively; the IDs 5S1 and 5R4 in the lichen samples correspond to *Cladonia* aff. *trassii* and a mixture of *Miriquidica instrata* and *Rhizocarpon geographicum* identified by our co-author Y. Ohmura, respectively (*M. instrata* is newly documented in Japan); the microbiome was analyzed in duplicate for all samples and given identifiers -1 and -2. The microbiomes were dominated by the phyla *Pseudomonadota* (6.2%–38.5%), *Acidobacteriota* (13.0%–30.0%), and *Bacteroidota* (6.3%–20.0%). Moreover, Pa and 5R4 showed a common feature of a high abundance (3.9%–7.1%) of the rarely cultivated phylum *Vulcanimicrobiota* [formerly *Candidatus* Eremiobacterota or WPS-2 (8, 9)], although the host organisms as isolation sources differed greatly. Thus, the data obtained in this study could serve as baseline data for comparison with microbiomes in similar environments or in efforts to cultivate uncultured microorganisms.

**TABLE 1:** Summary of microbiome amplicon data analyzed in this study

Sample name	Host species	Collection date (day/mo/yr)	No. of raw reads	No. of filtered reads	Biosample no.	Voucher specimens housed in the herbarium (TNS)
Plants						
As-1	*Artemisia sinanensis*	10/09/2022	39,260	24,455	SAMD00665337	Murai 1129
As-2			36,315	21,540	SAMD00665338	Murai 1129
Pp-1	*Parnassia palustris* var. *tenuis*	10/09/2022	39,784	22,663	SAMD00665339	Murai 1128
Pp-2			36,790	21,392	SAMD00665340	Murai 1128
Pa-1	*Phyllodoce aleutica*	10/09/2022	35,680	21,683	SAMD00665341	Murai 1123
Pa-2			36,013	22,331	SAMD00665342	Murai 1123
Lichens						
5S1-1	*Cladonia* aff. *trassii*	10/09/2022	74,906	46,213	SAMD00665359	Ohmura 14216
5S1-2			64,332	38,365	SAMD00665360	Ohmura 14216
5R4-1	A mixture of *Miriquidica instrata* and *Rhizocarpon geographicum*	10/09/2022	51,622	34,922	SAMD00665361	Ohmura 14221
5R4-2			44,488	30,811	SAMD00665362	Ohmura 14221

## Data Availability

The amplicon data set was deposited in the DDBJ Sequence Read Archive (DRA) under the accession number DRA017601 (BioProject/BioSample PRJDB17146/SAMD00665337–SAMD00665342, SAMD00665359–SAMD00665362).
